# Cave microbial communities are structured by environmental matrix and depth and can be characterized with field-portable assays

**DOI:** 10.1128/aem.00312-26

**Published:** 2026-03-23

**Authors:** Eric A. Weingarten, Brianna M. Fernando, Madelaine R. Freitas, Karl J. Indest

**Affiliations:** 1 Environmental Laboratory, U.S. Army Engineer Research and Development Center 199010 , Vicksburg, Mississippi, USA; 2 Cold Regions Research and Engineering Laboratory, U.S. Army Engineer Research and Development Center 102695 , Hanover, New Hampshire, USA; Colorado School of Mines, Golden, Colorado, USA

**Keywords:** microbial community structure, zoonotic pathogens, 16S rRNA gene sequencing, portable DNA extraction, environmental DNA (eDNA), subterranean ecosystems, cave microbiome

## Abstract

Terrestrial caves are unique ecosystems characterized by environmental stability, nutrient limitation, and absence of light, yet they host diverse microbial communities with ecological and public health relevance. Microbiome studies of caves have typically been limited in scope, sampling only select environments or cave depths. We conducted a broad survey of microbial diversity in three natural limestone caves and two abandoned mines spanning continental, subtropical, and arid climates in the United States. Using amplicon sequencing of 382 samples composed of soil, rock, water, air, and bat and rodent feces, microbiome composition was found to be primarily structured by cave location, followed by environmental matrix (soil, water, air, etc.), and transect distance from the cave entrance. Significant heterogeneity was observed both between and within caves, underscoring the need for spatially explicit and multi-matrix sampling to capture representative community profiles. Portable DNA extraction and quantitative PCR (qPCR) technologies for onsite detection of microbial pathogens were further validated, demonstrating comparable results to laboratory-based workflows and reducing sample-to-result turnaround time from several days to less than 2 h. Pathogen panels detected zoonotic and waterborne agents of human health concern, including *Salmonella* and *Legionella*, directly from cave environments. Collectively, our findings establish a methodological framework for robust microbiome characterization in subterranean ecosystems and highlight the feasibility of field-deployable genetic tools for both biodiversity mapping and rapid pathogen surveillance. These approaches will enable more systematic monitoring of cave environments, with applications for ecology, conservation, and public health.

**IMPORTANCE:**

Caves and mines represent extreme and isolated environments that harbor unique microbial communities, yet they remain among the least studied environments on Earth. Understanding how these communities are structured across different habitats and locations is essential for both ecological research and public health monitoring. In this study, we surveyed microbiomes across multiple caves and environmental materials to reveal how location, substrate type, and depth shape microbial diversity. We also demonstrated that portable DNA extraction and analysis tools can be used in the field to rapidly detect microorganisms, including potential pathogens, without the need for laboratory infrastructure. These results provide new insight into how microbial life is distributed in subterranean ecosystems and establish practical methods for monitoring microbial diversity and detecting pathogens in remote environments.

## INTRODUCTION

Natural terrestrial caves represent unique ecosystems that are characterized by nutrient limitations, stable temperature, and absence of light. Caves can be divided into different ecological zones depending on the level of light permeation, which often results in a trophic gradient (1, 2). For example, those areas close to the cave entrance and receiving regular direct light are referred to as the entrance and twilight zones and can be eutrophic in nature. Beyond the twilight zone, the transition zone receives only indirect light, followed by the dark zone which receives no sunlight and can be oligotrophic in nature and is characterized by stable temperature and humidity (latitude dependent). Despite these ecological constraints, caves can host transient and permanent fauna, including mammals, arthropods, and microbial communities that have adapted to these unique and often extreme conditions.

Despite a growing body of literature documenting the diversity and metabolic potential of cave microbial communities, the understanding of microbiome distribution, ecological roles, and variability across cave environments remains incomplete. Microorganisms function as primary producers in these environments, maintaining the nutrient cycle and obtaining energy from photosynthesis where light is available or chemolithoautotrophy in the dark (3–5). Previous studies characterizing cave microorganisms and microbial communities have largely focused on sampling unique geological formations like moonmilk and vermiculation deposits or different cave sample matrices, including rock, sediment, water, and air (6, 7). High-throughput DNA sequencing methods have provided extensive information on cave microbial community composition and diversity (8–16). Collectively, culture-dependent and -independent studies reveal that *Proteobacteria* and *Actinobacteria* are often abundant, and *Chloroflexi*, *Planctomycetes*, *Bacteroidetes*, *Firmicutes*, *Acidobacteria*, *Nitrospirae*, *Gemmatimonadetes*, and *Verrucomicrobia* also account for significant proportions of the microbial community in caves (14, 16–18).

Allochthonous microorganisms and nutrients transported by cave fauna like bats and rodents and by anthropogenic activities can further augment the cave microbiome and introduce microbial pathogens into these ecosystems. As a result, pathogenic agents responsible for histoplasmosis, rabies, leptospirosis, and tick-borne relapsing fever may be present in the cave microbiome, often introduced via animal scat (19–21). These agents sometimes cause disease in humans, and there are multiple reports of human-acquired histoplasmosis directly tied to cave exposure (20, 22). Furthermore, human leptospirosis has been tied to cave exposure, likely from contaminated water and/or animal waste (23).

Current understanding of microbial composition, diversity, and distribution patterns in terrestrial caves is limited due to minimal sampling regimes that fail to capture the spatial complexity and heterogeneity across the cave ecosystem. Separate studies have examined microbiome patterns by cave depth (24–26), region (27, 28), and sample type (29, 30), but we present herein a comprehensive method to capture complete microbiome zonation in subterranean systems. Biological sampling and biodiversity mapping of subterranean ecosystems require new strategies and methods for complex three-dimensional systems. In this study, sampling procedures and strategies were developed to facilitate baseline microbiome characterizations from multiple environmental matrices of subterranean environments from different cave systems. The sampling depth: number of samples, number of transects, and types of environmental matrices necessary to capture the complete microbial diversity of three cave systems and two abandoned mines using traditional amplicon sequencing were demonstrated. Also described herein is the effectiveness of emerging commercial solutions for field-portable DNA extraction and quantitative PCR (qPCR) for rapid detection of potential pathogens in caves. Clear differences in microbial composition by matrix, as well as along depth transects, were observed, underscoring the importance of sampling strategy in determining cave biodiversity and pathogen risk.

## MATERIALS AND METHODS

### Site selection and sampling procedure

Samples were collected from three natural cave systems and two abandoned mines between October of 2021 and August of 2022. Mammoth Cave (KY, USA), the longest cave system in the world, was sampled in two trips to a distance of 400 m from the historic entrance. Five soil samples were collected every 50 m. Water samples were collected from a waterfall at the cave entrance, from flowing water near the Mammoth Dome formation (~200 m from entrance) and from River Styx (~400 m). Duplicate air samples were collected at the cave entrance and at the farthest transect. A third air sample was obtained at both transects, but intentionally allowed mixing of suspended dust and water mist, which will be referred to as “composite” air samples. Two additional swabs of researchers’ clothing and shoes were taken after sampling was complete. Long Cave (KY, USA) is located less than 10 km from the entrance to Mammoth Cave, but is not known to connect to the main Mammoth Cave system and was sampled on the same days. Soil samples were collected every 50 m to a total distance of 250 m. Air samples were collected similarly to Mammoth Cave, with pure and composite samples taken at the entrance and farthest transect. Guano was collected from two seasonal bat roosts located on either side of a fork approximately 100 m from the entrance. At Hailes Cave located near Albany, NY, USA, soil swabs were collected every 15 m to a total distance of ~150 m. Air samples were collected at the entrance, middle transect, and farthest transect. Water samples were collected opportunistically following the path of a trickling stream. Two shallow, abandoned mines were sampled near Twentynine Palms, CA, USA, with soil collected every 5 m to total depths of 50 and 75 m for Duck Walk and Benchmark 19 Mines, respectively. Swabs of bare rock faces were collected separately from soil along the same transects. Feces were abundant in both mines, with apparent occupation by small rodents.

Samples were collected at Mammoth and Long caves with the assistance of National Park Service personnel under permit MACA-2021-SCI-0010. Hailes Cave sampling was conducted with the assistance of New York State Department of Environmental Conservation personnel. Sampling near Twentynine Palms, CA was conducted on DoD land by DoD personnel and did not require a permit.

For rock and soil, samples were collected with a sterile swab and transferred immediately to a 15 mL centrifuge tube containing 1 mL of RNAlater (Thermo Fisher Scientific, Waltham, MA). Guano was scooped directly into 50 mL centrifuge tubes. Water was collected either by submerging a sterile 1 L Nalgene bottle with a gloved hand when water was flowing or by filling the Nalgene bottle with a sterile syringe. Three models of air sampling devices were used: the Coriolis Compact (Bertin Technologies, Montigny-le-Bretonneux, France), which is dry cyclonic with a flow rate of 50 L/min; Coriolis Micro, which is liquid cyclonic with a flow rate of 300 L/min; and the AirPrep Bobcat (InnovaPrep, Drexel, MO), which uses a dry 0.01 μM air filter with a flow rate of 200 L/min. Each air sample was collected with the device at approximately chest height and run for 10 min. Separate laboratory and field testing showed no difference in amplifiable DNA collected between the devices (data not shown), so all results were treated equally. Composite air samples were collected by placing the sampler on the ground or near a water source and intentionally disturbing the environment to suspend dust and water vapor, with the device running for 10 min. To prevent sample contamination and transport of fungal spores outside the cave, researchers wore disposable Tyvek coveralls and gloves. All environmental samples were stored on ice until return to the laboratory in <24 h, where they were stored at −20°C until processed.

### DNA extraction and metabarcoding

Water samples were pre-processed by filtering them sequentially through 1.0, 0.45, 0.22, and 0.02 µm pore size filters. Filters were manually shredded with sterile scissors and transferred to Qiagen bead tubes. Air samples collected with dry devices were eluted in 25 mM Tris buffer. Eluted samples as well as samples collected with liquid devices were filtered through 0.22 µm filters and processed similarly to water. All samples were extracted with the DNeasy 96 PowerSoil Pro QIAcube HT Kit (Qiagen, Germantown, MD, Cat. #47021) following manufacturer instructions, with the addition of a 10 min heating step at 70°C prior to sample lysis. Metabarcoding targeted the V4 region of the 16S rRNA gene using the standard primers (515F/806R) recommended by the Earth Microbiome Project (31). Amplification was confirmed with 1.5% agarose gels, and amplicons were barcoded with Nextera XT Index kits (Illumina, San Diego, CA, Cat. #FC-131-2001). Barcoded libraries were purified with AMPure XP magnetic beads (Beckman Coulter, Brea, CA, Cat. #A63881), checked for quality with agarose gels, and quantified using the Quant-iT dsDNA HS Kit (Invitrogen, Waltham, MA, Cat. #Q33232). Libraries were normalized to 12 nM, pooled, and sequenced with the Illumina MiSeq platform using a 300-cycle reagent kit (Cat. #MS-102-2003) at a loading concentration of 8 pM with a 12% PhiX spike-in. Four separate sequencing runs were performed for the CA, NY, and two KY sampling trips.

### 16S data processing and statistical analysis

Illumina sequencing data were processed using *DADA2* (32). Separate analysis pipelines were performed for the four sequencing runs, with the amplicon sequence variant (ASV) tables merged prior to the detection and removal of chimeras. ASVs were classified against training set 18 of the Ribosomal Database Project (RDP) (33). Possible field and lab contaminants identified from extraction blanks were removed with the *decontam* package in R, which identified 545 potential contaminants out of 103,067 total ASVs (0.005%). Any samples with fewer than 500 reads were removed.

Sample coverage was calculated with the *phyloseq_coverage* function in the *metagMisc* package. Alpha diversity was calculated with the *estimate_richness* function in *phyloseq*, with ASVs observed used as the measure of species richness and the Shannon metric used to estimate diversity. Beta diversity was calculated with the *distance* function in *phyloseq* using Bray-Curtis distance and the ASV table converted to relative abundance. Dissimilarity was visualized with non-metric multidimensional scaling (NMDS) using the *metaMDS* command in *vegan*.

### Validation of field-portable DNA extraction protocol

The performance of the Qiagen PowerSoil Pro Kit was compared to the Biomeme M1 Sample Prep Kit (Biomeme, Philadelphia, PA), a field-portable purification kit that can process a sample in <2 min and which requires only a single disposable syringe. Initial testing included triplicate extractions of a commercial potting mix (PM) and soil from Long Cave, Mammoth Cave, and Benchmark 19 Mine and an extraction blank for each method. Extraction procedures included (Fig. S1): (i) Qiagen PowerSoil Pro Kit following the same methods as described above; (ii) M1 Kit with initial bead beating for 30 s using Qiagen bead tubes and CD1 solution using a Dremel (Fig. S2) with a custom tube adapter (CD1 + BB); (iii) M1 Kit with initial lysis using Qiagen bead tubes with Biomeme lysis buffer (BLB + BB); and (iv) M1 kits with raw, untreated soil. All extracts were quantified with the Quant-iT dsDNA HS Kit, checked for purity using 260/280 values from Nanodrop, and PCR amplified targeting the V4 16S rRNA gene to assess yield, purity, and amplifiability. We compared the sequencing results of Qiagen and M1 extractions with (CD1 + BB) and without homogenization (raw) of five soil samples from Mammoth Cave, five soil samples from Long Cave, two soil samples from Twentynine Palms, and three guano samples from Long Cave.

To assess the performance of field-portable DNA extraction used in conjunction with field-portable qPCR for pathogen detection, triplicate soil samples collected from each transect in Long Cave and three guano samples from a bat roost in Long Cave (*n* = 21 total samples) were extracted using Qiagen and M1 kits with homogenization (CD1 + BB) as an example data set.

### Validation of field-portable qPCR

We tested the performance of a field-portable real-time PCR device, the Biomeme Franklin, for rapid pathogen detection using lyophilized, shelf-stable reaction tubes. The panel included 11 targets and internal positive controls for bacteria and virus. Targets included six bacteria, two fungi, two parasitic protozoans, and one virus (Fig. S3). We tested pathogen presence in seven air samples, nine guano samples, one soil sample, and six water samples. Purified DNA (20 ng) was transferred to the reaction tubes, and DNA standards for each target were included at 10^7^, 10^5^, 10^4^, 10^3^, 10^2^, and 10^1^ copies, along with a no-template control.

DNA amplified by field-portable qPCR was sequenced and identified to validate that pathogen panels were amplifying the correct target by including one example each of a positive and negative result from field-collected samples, along with the 10^5^ and 10^2^ copy DNA standards for each target. Pathogen DNA was prepared with the Oxford Nanopore Ligation Sequencing Kit (Oxford Nanopore, Oxford, UK, Cat. #SQK-LSK109), multiplexed with the Native Barcoding Expansions (Cat. #EXP-NBD104; EXP-NBD114), and sequenced with an R9.4.1 MinION flow cell. Sequences were processed using EPI2ME software and the wf-16s workflow and aligned against the ncbi_16s_18s_28s_ITS database for taxonomic assignment.

## RESULTS

### Subterranean microbiome zonation by location, environmental matrix, and depth transect

**Fig 1:**
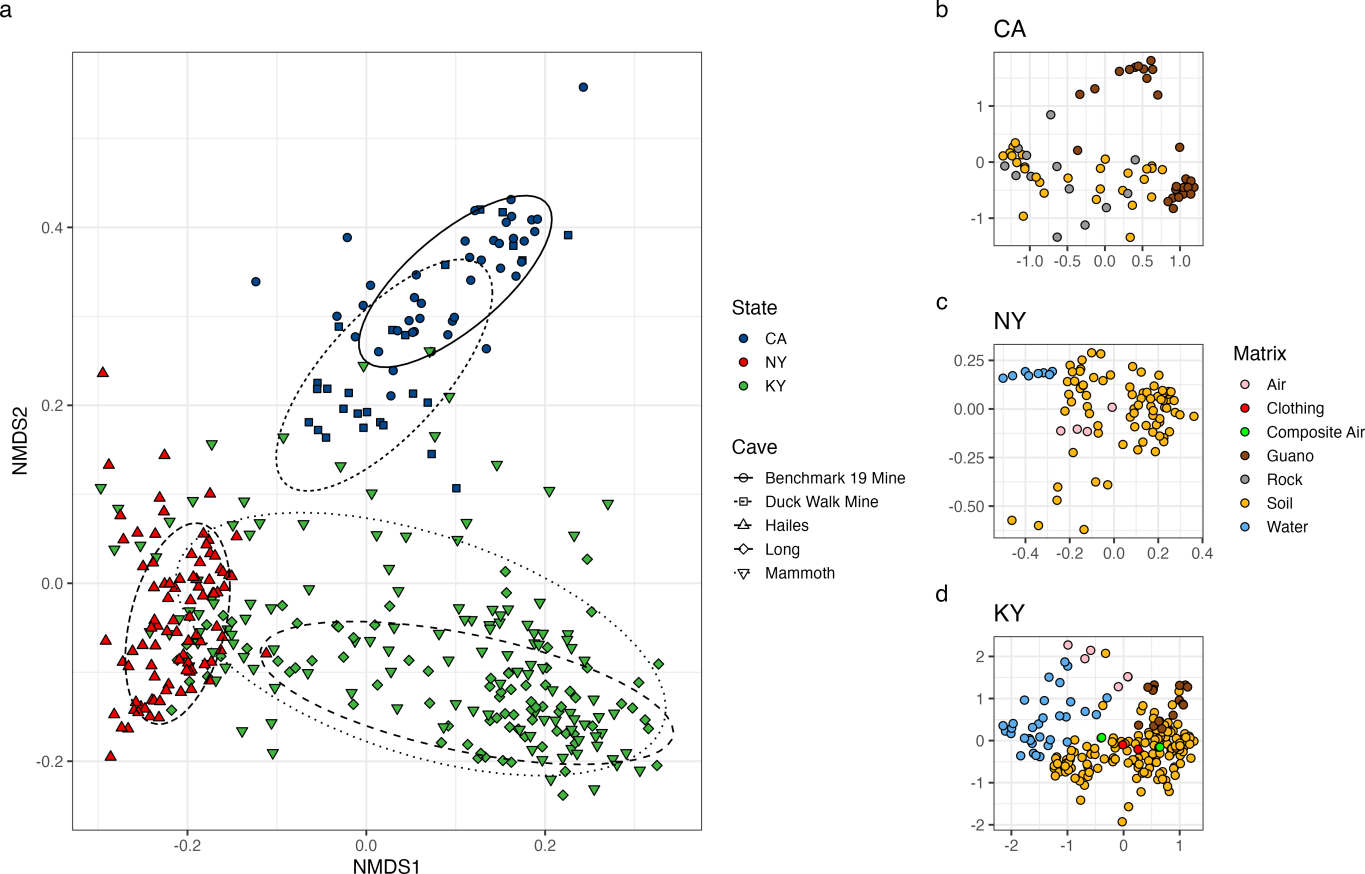
NMDS ordination of Bray-Curtis dissimilarity of all sequenced cave samples. Samples are grouped by location (state and cave) with ellipses surrounding 75% of samples belonging to each cave system (**a**). Samples are ordinated by environmental matrix for each state separately (**b–d**); CA panel contains both Benchmark 19 Mine and Duck Walk Mine, NY panel contains Hailes Cave, and KY panel contains Long Cave and Mammoth Cave.

**Fig 2:**
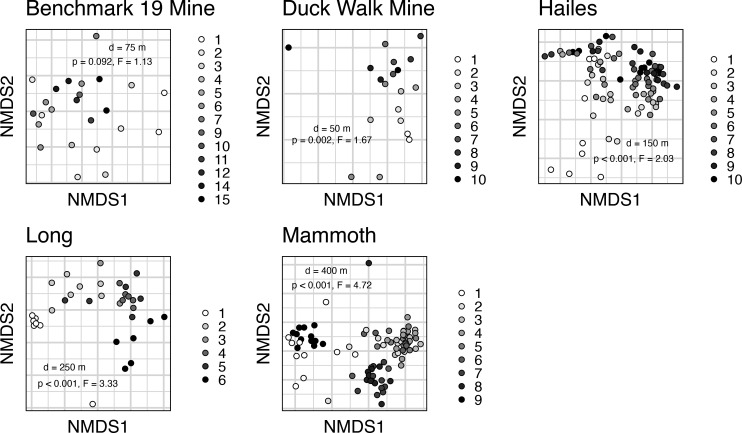
NMDS ordinations of Bray-Curtis dissimilarities of soil microbiota collected along depth transects in five subterranean systems. Total transect distance (d) is approximate linear distance from the cave opening to the farthest sample point. In each system, transect points were evenly spaced from the cave entrance to the interior. Separate PERMANOVAs were performed on each system, with transect as the independent variable and the distance matrix of 16S community data as the dependent variable. *P*-values and F-statistics of each PERMANOVA are inset in each ordination plot.

Sequence counts in the final data set of 382 cave samples ranged from 598 to 728,600 with a mean of 62,924. This corresponded to a range of sample coverage from 74.1 to 100% with a mean of 98.0%. Cave location (permutational multivariate analysis of variance [PERMANOVA], *P* < 0.001, *R*^2^ = 0.131) had the most significant impact on microbiome structure, with environmental matrix (*P* < 0.001, *R*^2^ = 0.059) having a secondary but still significant effect. NMDS ordination reflected this heterogeneity with clear separation between the taxonomic composition of caves in California, New York, and Kentucky (Fig. 1a). When the main effect of location was removed, and each site was ordinated separately, all pairwise comparisons of air, guano, soil, and water microbiome composition were significant (PERMANOVA, *P* ≤ 0.012, Fig. 1b through d). The only matrices that were similar in composition were soil and bare rock, which were collected separately from the California mine sites (*P* ≥ 0.150). In the Kentucky caves where composite air samples were collected, which intentionally contained suspended dust, composition was similar to both clean air and soil. The same was true for swabs taken from clothing after cave sampling was completed, representing an “average” composition of multiple environmental matrices. With only rock and soil samples retained, transect distance was a significant factor in community composition at all sites (PERMANOVA, *P* ≤ 0.002), except for Benchmark 19 Mine (*P* = 0.092). Total transect distance from the cave entrance ranged from ~50 to ~400 m, with composition changing along a consistent distance gradient (Fig. 2a through d). Mammoth Cave was the exception to this pattern, with composition instead grouping into three distinct clusters, with one cluster including both the nearest and furthest transect (Fig. 2e).

**Fig 3:**
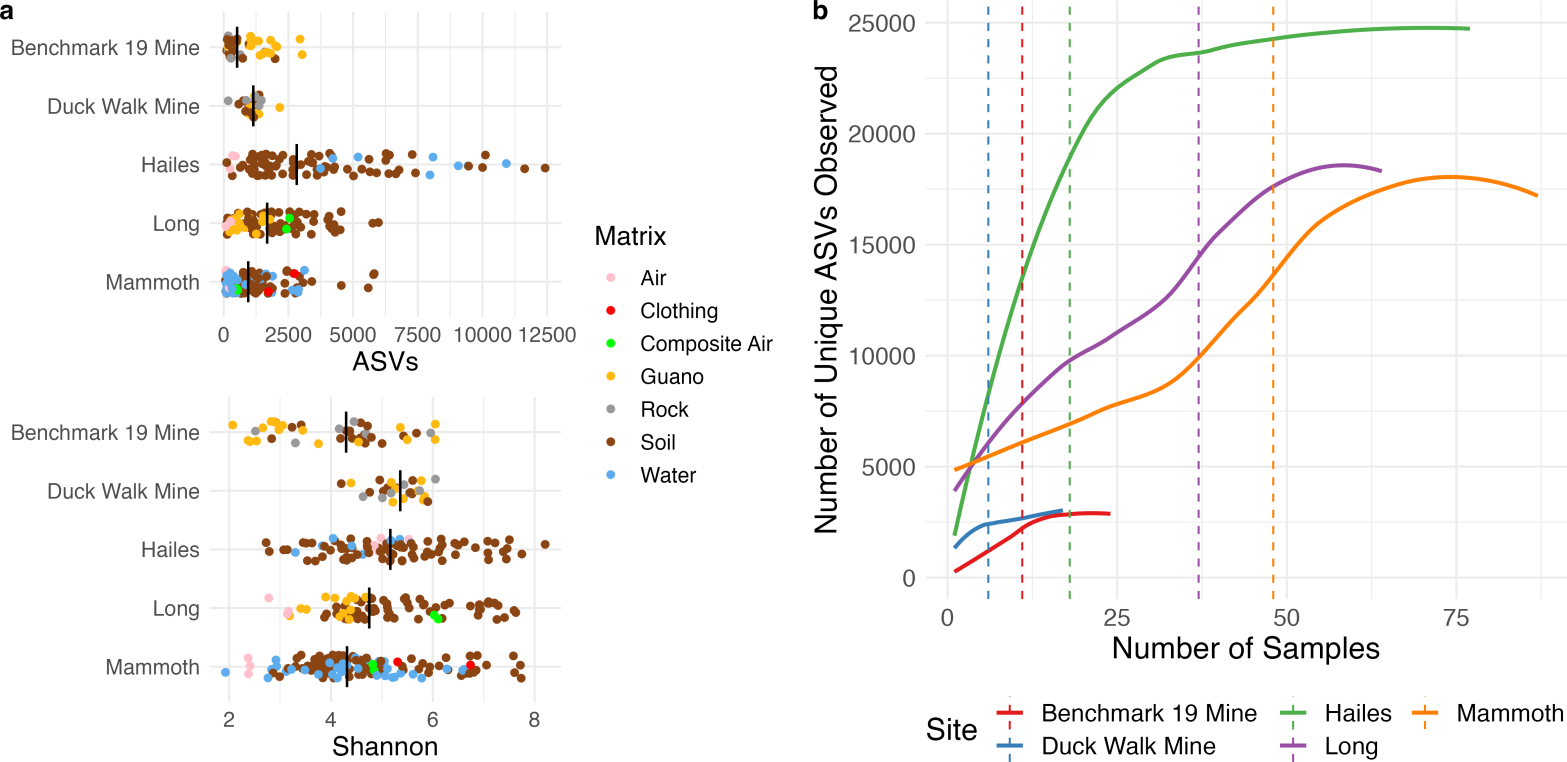
Alpha diversity of microbiota collected from each SubT system. Mean ASV total and Shannon diversity are marked with a vertical line, and individual samples are shown with points colored by environmental matrix (**a**). Rarefaction curves (**b**) show the number of unique ASVs identified by the total number of samples collected in each system separately. An 80% threshold of unique ASVs at each site is marked by a dashed line.

Similar to beta diversity, species richness and Shannon diversity were primarily determined by cave (multivariate analysis of variance [MANOVA], *P* < 0.001, *V* = 0.514), followed in variance explained by environmental matrix (*P* < 0.001, *V* = 0.183), and transect distance (*P* = 0.005, *V* = 0.033). Species richness in Hailes Cave was significantly greater than any other system (Tukey test, *P* < 0.001, Fig. 3a), while Shannon diversity was similar between Hailes and Long caves and Duck Walk Mine. Species richness did not differ significantly between any pair of matrices (*P* ≥ 0.072), but Shannon diversity was significantly higher in soil compared to both water (*P* = 0.045) and air (*P* = 0.005). Rarefaction of unique ASVs recovered from each SubT environment was used to determine the sampling intensity required to construct a representative census of the total microbiome. A total of 24,793 ASVs were identified between all rock and soil samples. A cutoff of 80% of all unique ASVs was used as a sampling threshold for each site. The two shallow mine sites, which had the lowest species richness generally, could be represented by 6 and 11 samples, while the natural caves required between 18 and 48 samples to be representative (Fig. 3b).

### Performance of field-portable DNA extraction protocol

Extractions of potting mix (PM) by Qiagen yielded an average of 71.1 ng/μL DNA, and soil from Mammoth Cave yielded 44.1 ng/μL. Neither Qiagen extraction of Long Cave or Benchmark 19 soil yielded measurable DNA. M1 extraction (CD1 + BB) of PM yielded 9.9 ng/μL and of Mammoth Cave soil yielded 0.2 ng/μL. As with Qiagen, extracts of Long Cave and Benchmark Mine yielded no measurable DNA. The other two M1 methods tested, BLB + BB and raw soil, yielded no detectable DNA for any sample type. To establish whether extraction yields were sufficient for PCR, we attempted to amplify the V4 16S rRNA gene of each test extraction, including extraction blanks and PCR positive and negative controls. Consistent with extraction yield, Qiagen extracts of PM and Mammoth Cave soil amplified successfully, while no replicates of Long Cave or Benchmark Mine soil amplified (Fig. S4). M1 (CD1 + BB), however, successfully amplified all replicates of Long Cave and two of the three replicates of Mammoth Cave while failing to amplify PM or Benchmark Mine. M1 (BLB + BB) failed to show any significant amplification. M1 with raw soil amplified only PM with some weak amplification of Long Cave soil.

**Fig 4:**
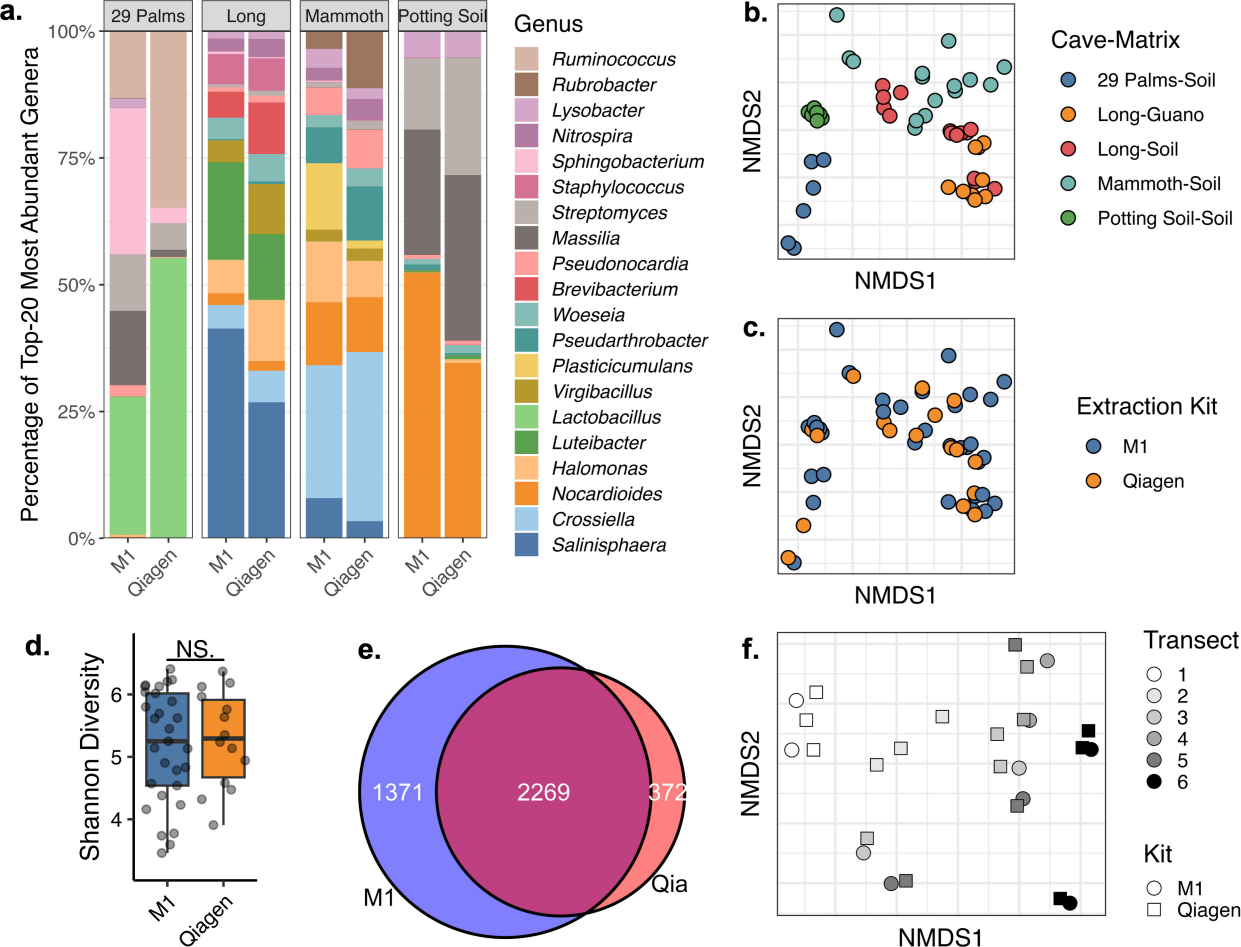
Comparison of 16S rRNA amplicon sequencing results obtained from lab-based Qiagen PowerSoil Pro kits and field-portable Biomeme M1 sample prep kits. (**a**) Genus-level diversity of soil collected from Twentynine Palms, CA, Long Cave, KY, Mammoth Cave, KY, and a commercial potting mix. (**b**) NMDS ordination of samples by cave and environmental matrix. (**c**) NMDS ordination of the same samples, with color representing extraction method. (**d**) Shannon diversity of M1 and Qiagen extractions. (**e**) Venn diagram representing the shared and unique ASVs obtained from M1 and Qiagen extractions of the same cave samples. (**f**) NMDS ordination of extractions by transect distance from the cave entrance and extraction kit. All plots represent information from the same samples. All NMDS ordinations represent Bray-Curtis dissimilarity.

Qiagen and Biomeme M1 extraction kits yielded very similar composition from amplicon sequencing of cave soil, mine soil, and commercial potting mix, even down to genus level (Fig. 4a). True environmental differences, including cave location (*P* < 0.001, *R*^2^ = 0.320) and environmental matrix (*P* < 0.001, *R*^2^ = 0.045), explained significant differences between the extractions (Fig. 4b), while the extraction methods themselves yielded almost no compositional difference (*P* = 0.989, *R*^2^ = 0.007, Fig. 4c). Likewise, Shannon diversity did not differ significantly between Qiagen and M1 extraction methods (Fig. 4d). Ignoring singletons, 57% of all ASVs were shared between Qiagen and M1 extractions (Fig. 4e), and more unique ASVs were observed from the M1 kit (34%) than from the Qiagen kit (9%).

We repeated amplicon sequencing of Qiagen and M1 extracts on soil and guano collected from all six transects at Long Cave (250 m) to determine whether M1 kits reproduced the previously observed distance effect on microbiome composition. As with comparisons of soil microbiota between the different sites, transect distance explained the majority of bacterial composition (*P* < 0.001, *R*^2^ = 0.464), while extraction method explained very little (*P* = 0.874, *R*^2^ = 0.018). NMDS ordination (Fig. 4f) showed that Qiagen and M1 methods performed equally well at separating transects by distance.

### Performance of field-portable qPCR for pathogen detection

Field portable Biomeme Go-Strip qPCR assays were first evaluated using the relevant spiked DNA controls in water, and all assays were able to achieve standard curves ranging from 5 to 6 logs and sensitivities ranging from 10^1^ to 10^5^ copies (Fig. S5). All 11 pathogen targets showed minimum detection of as low as 10^2^ copies. Correct target amplification was quickly confirmed for all six bacterial pathogens using alignment against NCBI using Oxford Nanopore EPI2ME software. The primary off-target identification was human DNA, and multiplex panels showed roughly equal amplification of each target (Fig. S6). EPI2ME was not able to align non-bacterial sequences, so manual alignment using BLAST and the core nucleotide database (core_nt) using the blastn algorithm was attempted for fungal, parasitic, and viral targets. *Pseudogymnoascus destructans*, *Histoplasma capsulatum*, *Giardia* spp., *and Cryptosporidium* spp. were correctly identified, while the *Rabies* assay aligned to an off-target *Klebsiella* phage.

Twenty-three environmental samples representing guano, water, air, and soil from Long Cave, KY, Hailes Cave, NY, and Twentynine Palms, CA were tested with the Go-Strip assays. Nine guano samples had 17 positive detections from 99 possible targets (17%); six water samples had nine positive detections from 66 possible targets (14%); seven air samples had nine positive detections from 77 possible targets (12%); and one soil sample had one positive detection from 11 possible targets (9%). Repeated positives included *Legionella* in Long Cave and Hailes Cave and *Salmonella* in Long Cave and at Twentynine Palms. The *Cryptosporidium* assay was positive for all samples tested, indicating a potentially high false-positive rate for that target. Plotting positive detections on a standard curve, *Legionella* copy numbers ranged from 100 to 60,000 copies (24.4 < Cq < 31.6), and *Salmonella* copy numbers ranged from 15 to 70,000 copies (23.9 < Cq < 34.2).

## DISCUSSION

Natural subterranean systems host diverse macro- and microbiological communities, with inhabitants often structured along a distance transect from the cave entrance to the deep interior (2). The cave microbiome is known to include viral, bacterial, and eukaryotic human pathogens, including zoonoses conferred by cave-dwelling animals, such as bats, rodents, and insects (19, 34). Investigation of cave microbiota and their potential threat to human health is challenging owing to the difficulty of access and sample collection in confined space. The aims of the research presented herein were to characterize cave microbiome heterogeneity between different caves, by depth within caves, and between different environmental matrices, as well as to validate field-portable DNA extraction and qPCR platforms for identifying potential pathogens. The availability of portable genetic analysis technologies will improve the understanding of cave biodiversity and may expedite disease detection, but their implementation will depend on the development of reproducible sampling protocols that encompass the many microhabitats in subterranean ecosystems.

We observed a primary effect of cave location, with apparent differences in microbiome composition between caves in CA, KY, and NY. Climate zone has been found to shape microbial diversity patterns in caves globally (27), and we identified a potentially similar effect, with highest diversity in NY, with a continental climate, intermediate diversity in KY, with a humid subtropical climate, and dramatically lower diversity in the hot desert climate of Twentynine Palms, CA. Alternatively, between-cave variability may have been driven by differences in the mineral composition of the cave surface, as has been observed in gypsum caves (35). Secondary to the between-cave effect, we observed differences in microbiome composition between air, guano, rock, soil, and water matrices within each cave sampled. Turrini et al. (36) reviewed the effects of airflow, water movement, and animals, which move between the cave and aboveground (troglophiles) for transporting scarce organic material into the cave. We observed the greatest compositional difference between endogenous (rock, soil) and exogenous (air, guano, water) cave material, potentially owing to matrix connectivity to the aboveground environment. Flowing and percolating water are the primary sources of exogenous organic material in caves (37), and we found surprisingly high richness and diversity of water-borne microbiota relative to soil organisms. There was relatively little difference in composition between soil and bare rock, and what difference was observed is likely also related to nutrient availability arising from the process of weathering (24). We found distance from the cave entrance to be a tertiary driver of composition, a process that is well described in both subterranean and aquatic ecosystems and also related to the availability of light and organic material (2).

Focusing on within-cave heterogeneity, the finding of significant distance and matrix effects has obvious implications for cave exploration for biodiversity and disease surveillance. We have demonstrated that a representative snapshot of the cave microbiome cannot be achieved from sampling a single matrix, or even from a single point in a cave. Hailes Cave, NY contained the highest species richness, but rarefaction actually found that Long and Mammoth caves required two to three times more samples to achieve 80% sample coverage. This is likely a result, in part, from sampling the KY caves to depths of 250 and 400 m compared to only 150 m in NY. Unlike other sampling expeditions to similar distances (25, 38), we found no “normalizing” of the microbiome beyond the light zone and instead found continuous compositional change throughout the route of entry. Tourist caves often contain greater species richness (39), and it is notable that Mammoth Cave is among the most visited in the world, but microbial import from bats may similarly supplement the cave microbiome (40), and both Long Cave and Hailes Cave are overwintering sites for thousands of bats. We hypothesize that the most likely explanation for the distance-diversity relationship we observed is the degree of branching in a cave’s morphology. The section of Hailes Cave we explored was linear and unbranched; Long Cave had a single split; and Mammoth Cave contained many branches. Microclimatic differences, even between individual chambers, can be substantial and are known to drive microbiome differences (41). Furthermore, built features, such as artificial light, can modify microbial composition (28), and this was likely true in at least Mammoth Cave. This leads to the conclusion that any attempt to fully survey a cave’s microbiome must include frequent sampling events along the passage of interest to the observer.

Biological survey of caves has attracted interest for disease surveillance (19), endangered and invasive species monitoring (42, 43), natural products discovery (44), and exploration of other planets (45), among other applications. Systematic investigation of these topics will benefit from advances in robotics (46–48) and autonomous sample collection (49, 50) to allow greater access and sampling frequency than could be achieved by individual investigators. These technologies may be particularly important for capturing the microbiota of different environmental matrices. Composite sampling of the air, focused on commercially available air samplers (51), may be an adaptable engineering approach for autonomous cave surveillance with minimal sampling effort. It was determined that air sample collection for 10 min was sufficient for capturing the cave aeromicrobiome, and intentional inclusion of mist and dust from the surrounding environment accurately represented a composite of each matrix individually.

The utility of field-portable DNA extraction methods was evaluated to complement interest in rapid, onsite microbial diversity and pathogen surveillance. Portable “field laboratories” have been used for point-of-care diagnostics and environmental DNA monitoring (52, 53), and a miniaturized lab case the size of airline check-in luggage based around the Biomeme M1 sample prep kit was developed. The Biomeme M1 kit has been used primarily in clinical surveillance (54–56), but it has been demonstrated here to be effective for PCR-based environmental microbiome analyses. DNA extraction yields were found to be inferior to laboratory-based Qiagen PowerSoil extractions, as observed with other field-portable kits (57), but yield and purity were sufficient for amplicon library prep and qPCR from all samples tested. A manual cell lysis step using bead beating was determined to be critical to achieving useful extractions, but this can be achieved in the field with a Dremel tool (58). Critically, sequencing of M1 and Qiagen extracts showed nearly identical community composition and diversity, and the portable M1 kit was able to independently reproduce the effects of cave location, environmental matrix, and transect distance. The full mobile extraction protocol consumes less than 5 min per sample, and products are immediately ready for PCR.

The objective of the work presented here was to pair rapid sample collection and extraction with onsite qPCR detection of a suite of known cave pathogens as a proof-of-concept for in-cave biological threat identification. The pre-mixed and shelf-stable Biomeme Go-Strip qPCR panel targeted zoonotic and water-borne diseases known to occur in caves (*H. capsulatum* [59], *L. interrogans* [60], *R. lyssavirus* [61], *S. enterica* [62], *Legionella* spp. [63], *E. coli* [64], *L. monocytogenes* [65], *Giardia* spp. [66], *Campylobacter* spp*.* [67], *Cryptosporidium* spp. [68]). Spike-in control experiments showed that all 11 targets on our panel were detectable by qPCR from less than 10^2^ total gene copies and from as few as 10^1^ copies, which are commonly used thresholds for detecting low-abundance pathogens from environmental samples (69, 70). Critically for the prospect of employing qPCR for in-cave pathogen monitoring, the full sample collection to positive/negative identification could be performed in less than 2 h for each target. The Biomeme assays can be multiplexed with three targets per sample well, enabling screening of up to nine targets at a time from up to three environmental samples or three targets at a time from nine samples, depending on user configuration (71). We detected 36 pathogen “hits” from 253 possible targets from real cave samples (14% positive). All Cq values for positive identifications were <35, a threshold used in cave disease surveillance previously (72), and all negative controls had no Cq value, meaning results from real-world samples were consistent with Environmental Microbiology Minimum Information (EMMI) guidelines (73). Together, these results demonstrate that field-portable qPCR can provide rapid, multiplexed, and standardized pathogen surveillance directly within the cave environment.

This study represents the largest synthesis of cave microbial ecology with emerging techniques for field-deployable environmental genetics and pathogen monitoring to our knowledge. High spatial and matrix heterogeneity in microbiomes of caves from continental, subtropical, and arid climates was found, underscoring the need to collect genetic material throughout cave passageways to develop representative data sets. Several commercial options exist for this, and it is proposed that air sampling may be the most robust approach currently available. We show that nucleic acid extraction performed on the same samples in a state-of-the-art environmental genetics lab and in the field from a single Pelican case produced statistically similar results using qPCR and sequencing approaches. Sample processing time was reduced from days to under 2 h. It may be possible to further accelerate targeted genetic surveillance of caves with rapid PCR devices based on magnetic induction cycling (74) and loop-mediated isothermal amplification (LAMP) (75), but such approaches are likely to remain limited to previously known targets numbering in the dozens per experiment. Semi-targeted or target-agnostic sequencing of any organism, including emerging pathogens, using Nanopore sequencing is a likely improvement on PCR-based surveillance (76, 77). We demonstrated that the products of qPCR reactions could be directly sequenced with a MinION device in a field setting without a cleanup step, offering the possibility of orthogonal confirmation of target detection. Existing products for sample collection, PCR, and sequencing performed well in the cave environment, but nucleic acid extraction, library prep, and downstream bioinformatic analysis were identified as the primary bottlenecks to field-deployable genetic surveillance in caves, warranting additional study and commercial development.

### Conclusion

This work establishes both a methodological framework and a technological proof-of-concept for comprehensive cave microbiome and pathogen surveillance. By demonstrating that cave microbial communities are shaped in order of influence, by location, matrix material, and distance transect, the necessity of spatially explicit and multi-matrix sampling strategies to capture the full extent of subterranean biodiversity was highlighted. It was further shown that commercially available, field-portable extraction and qPCR technologies can generate results comparable to laboratory-based methods, with the benefit of near real-time species detection. Together, these findings advance the study of cave microbial ecology, provide scalable approaches for biodiversity mapping and disease surveillance, and provide a foundation for future integration of autonomous sampling and sequencing technologies to monitor subterranean environments more effectively.

## Data Availability

The 16S amplicon sequencing data are available in the NCBI Sequence Read Archive (SRA) under BioProject PRJNA1419905.

## SUPPLEMENTAL MATERIAL

Supplemental material aem.00312-26-s0001.docx Figures S1 to S6.

ASM does not own the copyrights to Supplemental Material that may be linked to, or accessed through, an article. The authors have granted ASM a non-exclusive, world-wide license to publish the Supplemental Material files. Please contact the corresponding author directly for reuse.
