# Supplementary material for: Cave microbial communities are structured by environmental matrix and depth and can be characterized with field-portable assays
Source: Appl Environ Microbiol. 2026 Mar 23;92(4):e00312-26. doi: 10.1128/aem.00312-26 (PMC13101471; doi:10.1128/aem.00312-26)
Supplement: Supplemental material — Figures S1 to S6. [file aem.00312-26-s0001.docx]

**Cave Microbial Communities are Structured by Environmental Matrix and Depth and can be Characterized with Field-Portable Assays**

Eric A. Weingarten^a^, Brianna M. Fernando^b^, Madelaine R. Freitas^a^, and Karl J. Indest^a^

^a^Environmental Laboratory, U.S. Army Engineer Research and Development Center, Vicksburg, MS, USA 39180

^b^Cold Regions Research and Engineering Laboratory, U.S. Army Engineer Research and Development Center, Hanover, NH, USA 03755

**Supplementary Information**

Fig. S1. Schematic of tested DNA extraction protocols. Pipelines included (i) standard Qiagen PowerSoil Pro laboratory kit, (ii) Biomeme M1 field portable extraction kit with sample homogenization in Qiagen lysis buffer, (iii) Biomeme extraction kit with sample homogenization in Biomeme lysis buffer, and (iv) Biomeme extraction kit with no sample homogenization.


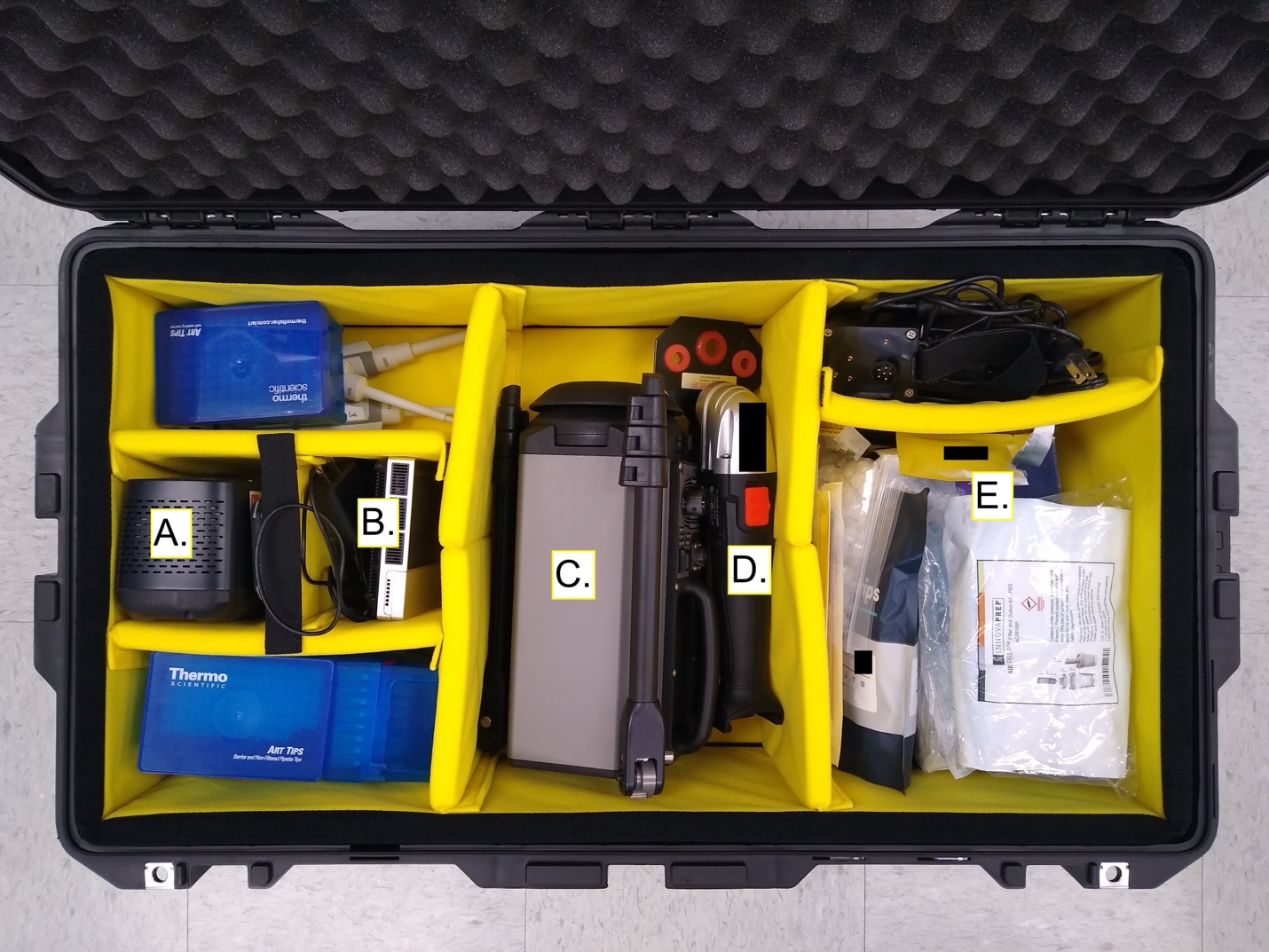


Fig S2. Photograph of the field-portable DNA extraction and analysis lab used in the study, contained within a 75.2 x 39.4 x 23.8 cm Pelican 1615 Air Case. Contents include: (a) Biomeme Franklin qPCR thermocycler, (b) Oxford Nanopore MinION Mk1C sequencer, (c) InnovaPrep Bobcat air sampler, (d) BioSpec SoniBeast-JR bead disruptor, and (e) packaged air sample collection kits, M1 sample prep kits, and Biomeme Go-Strips.

Fig S3. Schematic of the lyophilized, field-portable Biomeme Go-Strip panels designed for the study. Each well of the qPCR assay can identify three unique targets with the use of FAM, ATTO 647N, or Texas Red-X dyes. The two panels constructed targeted six bacterial pathogens (*Campylobacter spp., Escherichia coli, Legionella spp., Leptospira interrogans, Listeria monocytogenes,* and *Salmonella spp.*), two fungal pathogens (*Histoplasma capsulatum* and *Pseudogymnoascus destructans*, the species responsible for white-nose syndrome), two parasites (*Cryptosporidium spp.* and *Giardia spp.*), and the *Rabies* virus.

Fig S4. Gel images of PCR amplifications of the V4 16S rRNA gene from extractions of commercial potting mix (PM) and soil from Long and Mammoth Caves and Benchmark 19 Mine. Each gel contains a 100-1000 bp ladder and the first gel contains a PCR positive control (+) and negative control (-). The extraction methods included: (i) Qiagen PowerSoil Pro; (ii) Biomeme M1 kit with upstream bead beating in Qiagen CD1 solution (CD1+BB); (iii) M1 kit with upstream bead beating in Biomeme Lysis Buffer (BLB+BB); and (iv) M1 kit with raw soil that was not homogenized. Each extraction method included an extraction blank (B).

Fig S5. Standard curves derived for all pathogen targets detected by DNA with custom Go-Strip qPCR panel. Spike-in DNA standard concentrations were 10^7^, 10^5^, 10^4^, 10^3^, 10^2^, and 10 copies. DNA copy number is displayed on the x-axis and Ct values are displayed on the y-axis.


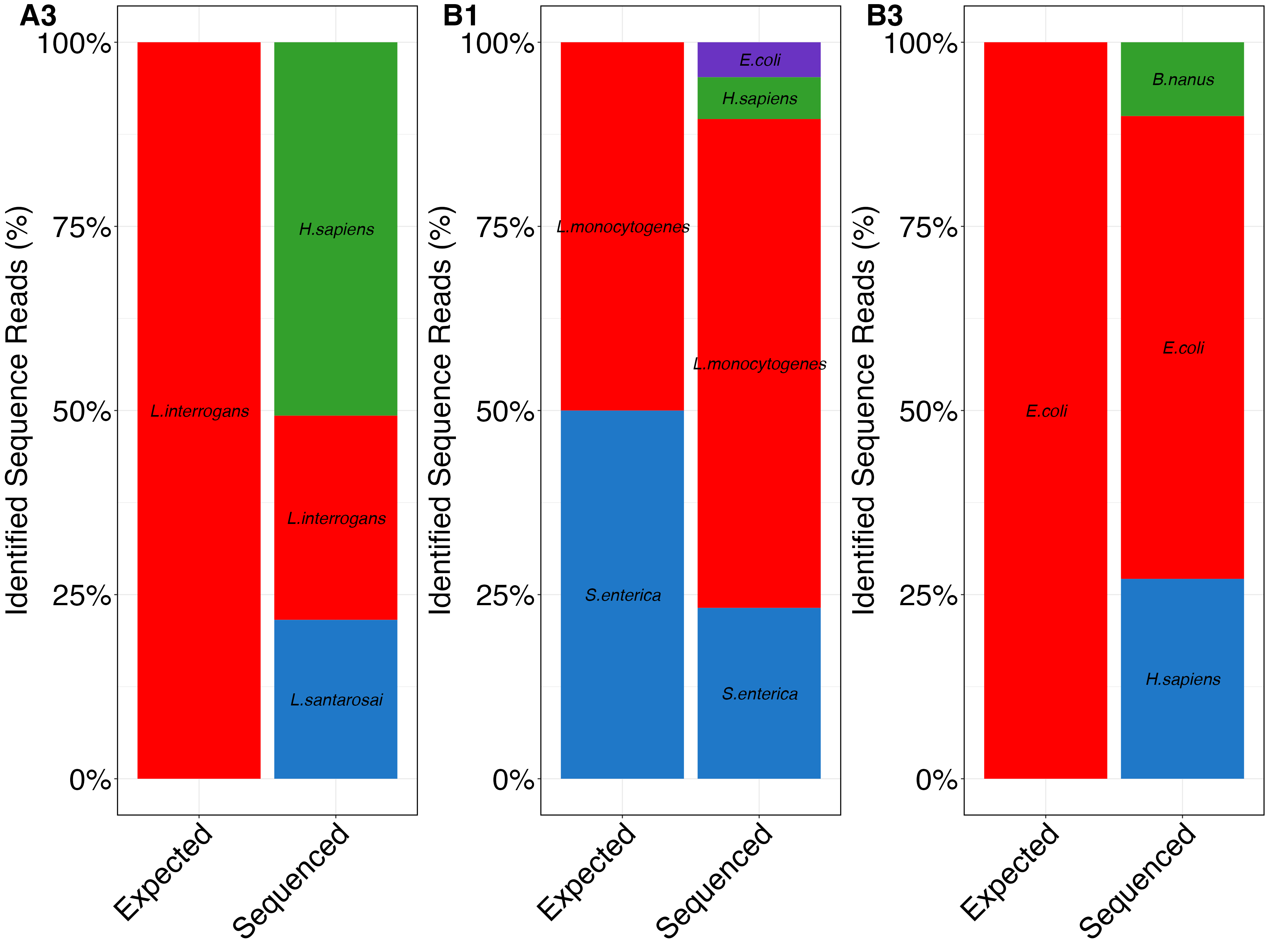


Fig S6. Oxford Nanopore sequence identities obtained for Go-Strip panels targeting bacterial pathogens. The letter in the label identifies either Go-Strip panel A or B. The number in the label identifies the corresponding well of the Go-Strip panel. On the left of each panel is the target composition and the right is the species-level identity determined by sequencing the qPCR product of each assay.
